# Are human resource managers with good listening competency more likely to avoid job burnout?

**DOI:** 10.1186/s12889-022-12618-x

**Published:** 2022-02-07

**Authors:** Yanqing Wang, Hong Chen

**Affiliations:** 1 School of Economics and Management, China University of Mining and Technology, Xuzhou, Jiangsu Province China; 2 School of Business, Jiangnan University, Wuxi, Jiangsu Province China; 3 Institute for National Security and Green Development, Jiangnan University, Wuxi, Jiangsu Province China

**Keywords:** Human resource manager, Job burnout, Listening competency, Role stress

## Abstract

**Background:**

Listening is an important responsibilities of human resource managers, whether it will bring role stress to human resource managers, or lead to the risk of job burnout. This study aims to analyze the impact of listening competency on job burnout among human resource managers, and examine the mediating effect of role stress.

**Methods:**

This study adopted a cross-sectional method to randomly select 500 human resource managers from China’s top ten human resource management cities to conduct an online questionnaire survey, and 232 valid samples were obtained. Descriptive statistical and one-way ANOVA were used to explore the status of job burnout among human resource managers in China. Correlation analysis, multiple linear regression and mediating effect analysis were employed to test the relationship between listening competency and job burnout, as well as the mediating effect of role stress.

**Results:**

(1) 34.5% of the respondents reported mild burnout, while 3.0% respondents showed serious burnout. Emotional exhaustion was the most serious. (2) Those are good at listening could easily avoid job burnout. Among them, listening skills were conducive to reducing the degree of depersonalization of human resource managers, and empathy was more conducive to improving their personal sense of accomplishment. (3) The role stress had a significant mediating role in the relationship between listening competency and job burnout. Which means that listening competency can avoid job burnout by reducing role stress of human resource managers.

**Conclusions:**

This study revealed the current situation of job burnout among human resource managers in China, and explored the influence of listening competency on job burnout. This study enriched the research content of job burnout, and provided references for preventing and intervening job burnout of human resource managers.

## Background

Job burnout is a state of physical and emotional consumption of workers due to the long-term stress work environment [1–3], and also a syndrome caused by lack of resources or incentives [4], and includes three components: emotional exhaustion, depersonalization, and reduced personal deprivation [5]. The harm from job burnout is multifaceted. It not only leads to a reduction in career crises, such as reduced work efficiency, job satisfaction, and turnover [6–8], but also exposes individuals to physiological diseases, such as hypertension, cardiovascular disease, and sleep problems [9]. For example, studies have shown that individuals with job burnout experience are more likely to have cognitive difficulties [10]. A meta-analysis showed that the global job burnout rate of residents was 51.00%, In particular, 51.64% in North America, 22.72% in Europe, and the highest level in Asia was 57.18% [11]. Another study found that 80.86% of miners had different degrees of job burnout, among which the proportion of moderate or severe job burnout was as high as 46.41% [12]. The 2017 “*Report on Occupational Mental Health of Employees in China*” showed that 28.85% of employees in the workplace suffered from job burnout [13]. Job burnout has evolved into an occupationally harmful phenomenon that has a wide impact on both physical and mental health [12]. how to effectively deal with job burnout has become a major challenge faced by various organizations.

HR managers are those who work in human resource planning, employee recruitment and selection, performance appraisal, salary and welfare management, incentive and training, and labor relationship coordination. They are required to have good communication skills and be able to coordinate various relationships within the organizations. In addition, HR managers are often regarded as managers of job burnout, so HR managers’ job burnout will not only affect their own physical and mental health, but also directly affect other employees and even organizational performance. Kan (2014) [14] found that the job burnout of HR managers in China was of the middle level, but little concern existed regarding job burnout of HR managers and its influencing factors. Considering that preventing and reducing job burnout of HR managers is vital important to improving their own physical and mental health, and forms the basis for ensuring the sound development of organizations. Therefore, the current study aims to investigate the state of the job burnout among HR managers in China and explore possible approaches to avoid it.

### Listening competency and its role in the workplace: what does the current evidence say?

Listening is a basic but complex phenomenon in interpersonal communication and it’s also an important part of the intersection theory and ethical care [15]. Castro et al. (2018) [16] defined listening as a behavior that showed concern, understanding and good intention toward the speaker. In a trust and openness atmosphere, listening sends a signal to the speaker that listener is engaged in continuous cognitive processing. Good listening behavior promotes the psychological security level of subordinates [17]. Individuals who are good at listening are more likely to gain the trust of others [18], establish good interpersonal relationships, and promote teamwork [19], thus helping improve work performance. Brenner (2017) [20] showed that listening is at the heart of effective psychiatric practice and provides a foundation for many of the established competencies in psychiatry training. Drollinger and Comer (2013) [21] indicated that salesperson’s listening competency as an antecedent to relationship selling. The perception of good listening is positively associated with job satisfaction, relationship satisfaction, better emotions and lower job burnout [22]. Roche and Ogden (2017) [23] studied the job burnout and the health status of Samaritans’ listening volunteers, the results showed that listening volunteers exhibited lower job burnout and good health. However, none of the studies examined the relationship between listening competency and job burnout among HR managers. According to the Job-Demand Resource model (JD-R) which indicates that personal resources can be regarded as a kind of job resources [24] and an increase in individual resources is conducive to reducing the job burnout [25]. Listening competency can also be regarded as an important personal resource for HR managers, and future research is warranted to test whether good listening competency can help HR managers avoid or reduce job burnout.

### Role stress: a potential mediator between listening competency and job burnout?

Role stress is an important part of work pressure [6]. Role stress occurs when external role expectations exceed employees’ abilities and they are unable to complete their work tasks [26, 27]. Generally, role stress includes three components: role conflict, role ambiguity, and role overload [28, 29]. Role overload can be further divided into role quality overload and role quantity overload. The role quality overload refers to the complexity or difficulty of work tasks exceeding the employee’s ability scope, and role quantity overload refers to the employees that are unable to complete too many work tasks at the same time [29, 30]. HR managers have to not only carry out the orders of leaders to safeguard the interests of the organization but also provide employees with necessary job security to protect their rights. So they often face the difficulties such as balance of interests between organization and employees, management and services, and role conflicts of HR managers in the organization have become the main source of their anxiety and stress. Besides, HR management in China is still in the transitional stage, the white paper “*Survival and Development Status of China’s Human Resource in 2019*” (released by hrloo.com, a professional HR communication platform) pointed out that only 26% HR managers received a professional HR management training and the work boundaries of Chinese enterprises are not clear between personnel and administrative. So the low level of specialization in HR management, role conflict, and unclear job responsibilities have significantly increased the role stress of HR managers.

A large number of empirical studies have shown that role stress has a significant positive impact on job burnout [31–33]. In addition, Pecino et al. (2019) [34] confirmed that a positive organizational climate could lead to less role stress and burnout out workers. Kilroy et al. (2016) [35] found that role conflict and role overload partially mediated the relationship between high-involvement work practices and job burnout. However, few studies have examined role stress as a mediator between job burnout and its avoidance factors. This leaves the potential mechanisms of change, such as the mediating role of role stress, unexamined and future studies are warranted to assess how burnout is reduced. Good listening competency can help HR managers grasp the real needs of employees, so as to better balance the interests between enterprises and employees, as well as reduce conflicts. In addition, the majority of formal and informal learning takes place while one is listening. In other words, people acquire most of their knowledge throughout their life through listening [36]. In this respect, good listening competency can help HR managers improve their knowledge and skills, which can help them to clarify their responsibilities, decrease unnecessary resource consumption, and also improve their work efficiency. Thus, future research is warranted to test whether listening competency can reduce HR manager’ role stress, and so as to reduce their job burnout.

### Current study

**Fig. 1:**
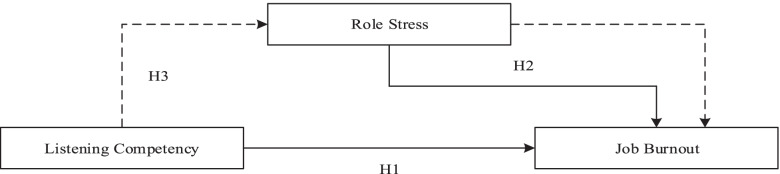
Theoretical model underlying empirical research

This study contributes to the current literature on the impact of listening competency and role stress on job burnout among HR managers, and has important references for preventing and intervening job burnout of HR managers. Figure 1 showed the conceptual model of this study. Based on the evidence reviewed above, the main purpose of the current study is to evaluate the impact of listening competency on HR managers’ job burnout. We also aim to evaluate the impact of role stress on HR manager’ job burnout and the mediating role of role stress in the relationship between listening competency and job burnout. Specifically, we proposed three hypotheses:

- Hypothesis 1 HR managers’ listening competency is significantly negatively correlated with job burnout.
- Hypothesis 2 The role stress of HR managers is positively correlated with their job burnout.
- Hypothesis 3 Role stress has a significant mediating effect on the relationship between listening competency and job burnout of HR managers.

## Methods

### Study design and participants

This study was conducted in the top ten HRs cities in China (e.g. Beijing, Shanghai, Wuhan, Nanjing, Suzhou, Hangzhou, Guangzhou, Shenzhen, Chengdu, and Chongqing). And HR managers working on the Internet, real estate, manufacturing, finance, and service industries were included as the main survey participants. Then, an online survey method was adopted, and questionnaires were distributed one-to-one through WeChat (the largest social networking platform in China), with the same reliability and validity as traditional paper-and-pencil questionnaires [37]. Before the survey, it was confirmed that volunteers could participate and fill out the questionnaire at any time and in any place. A total of 500 questionnaires were distributed in this study, and 268 questionnaires were collected. After eliminating the invalid samples, finally 232 effective samples were obtained, and the effective recovery reached 86.57%. Table 1 showed the structure of sample distribution.

**Table 1:** Demographic characteristic of the sample (*N* = 232)

Demographic		Proportion	Demographic		Proportion
Gender	Male	54%	The nature of organization	State-owned enterprise	29%
	Female	46%		Private enterprise	51%
Age	21 to 30	62%		Sino foreign joint venture	4%
	31 to 40	35%		Foreign-Funded enterprise	4%
	Above 40	3%		Others	12%
Marital status	Married	43%	Number of Employees	Less than 20	10%
	Spinsterhood	57%		20 to 100	14%
Diploma level	Junior College	8%		100 to 300	17%
	Bachelor’s Degree	74%		300 to 1000	20%
	Master’s Degree	18%		1000 to 2000	11%
Social work age (Year)	Less than 1	15%		Above 2000	28%
	1 to 3	26%	Positional Hierarchy	HR specialist	38%
	3 to 5	16%		HR supervisor	21%
	5 to 10	24%		HR manager	20%
	10 to 15	14%		HR director	4%
	Above 15	5%		HR business partner	6%
Yearly income (1000¥)	Less than 30	12%		Others	11%
	30 to 80	22%			
	80 to 150	39%			
	150 to 300	18%			
	Above 300	9%			

### Instruments

#### Job burnout

When measuring job burnout, the MBI-GS was used [38], which was known as the gold standard for measuring job burnout [39], which also has good reliability and validity in a variety of occupational and cross-cultural researches [40]. Therefore, MBI-GS can be used to measure the job burnout of Chinese HR managers. This scale had a total of 15 questions, including three dimensions of emotional exhaustion, depersonalization, and reduced personal accomplishment. The Likert 7-point scoring method was adopted (0 = “never” and 6 = “every day”). Items with reduced personal accomplishment were scored in reverse. In this study, the Cronbach’s α coefficient of the three dimensions was 0.87, 0.82, and 0.86, respectively.

#### Listening competency

**Fig. 2:**
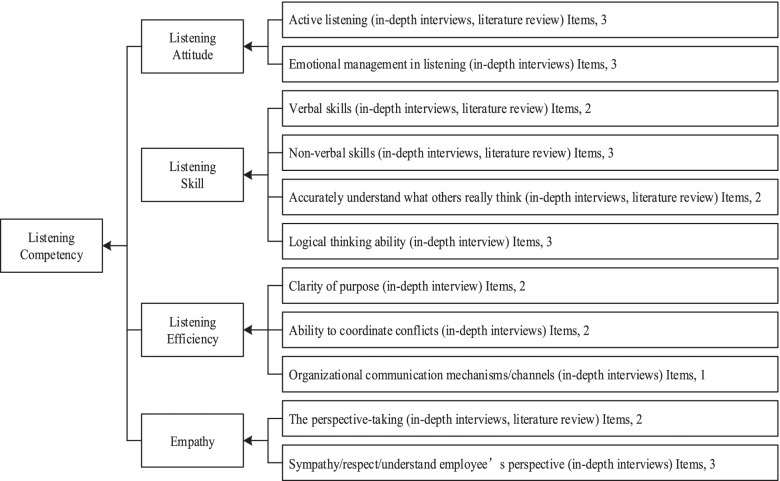
The structure of listening competency scale

In the existing studies, listening competency was mostly measured based on the respondents’ perception [41] and focused on listening competency of practitioners in specific industries, such as medical and sales staff [42–44]. Listening has also appeared in the form of multidimensional variables in previous studies. This study referred to the research of Gao et al. (2012) [45]. Based on the grounded theory, using a qualitative analysis method, and combining relevant literatures, listening competency was subdivided into four dimensions in this study: listening attitude, listening skill, listening efficiency, and empathy (Fig. 2 showed the structure of listening competency scale). On this basis, this study independently developed the listening competency scale, which contained 26 items, and adopted Likert’s 7-level scoring method (1 = “completely disagree” and 7 = “fully conform”).

This study adopted the self-developed scale for measuring listening competency. The reliability and validity of the questionnaire were analyzed through pre-testing, and a formal questionnaire was formed after correcting the 21 questions included. In the formal survey, Amos 22.0 was used to conduct a confirmatory factor analysis of the scale (*χ2/df = 2.103; RMR = 0.043; GFI = 0.866; CFI = 0.948; NFI = 0.906; and TLI = 0.939*). The results showed that the overall fitting effect of the model was good. In this study, Cronbach’s α coefficient of the four dimensions of listening competency was 0.90, 0.91, 0.80, and 0.90, respectively.

#### Role stress

When measuring the role pressure, this study adopted the role stress scale from Chen (2013) [46], which consisted of four dimensions: role conflict (6 items), role ambiguity (6 items), role quality overload (5 items), and role quantity overload (5 items). The role stress scale was further revised through pre-testing, and finally 15 questions were included, all of which were scored using the Likert 7-point scoring method (1 = “completely inconsistent” and 7 = “completely consistent”). In this study, the Cronbach’s α coefficient of the four dimensions was 0.77, 0.72, 0.88, and 0.77, respectively.

Socio-Demographic Data: Previous studies have shown that demographic variables (such as gender, social work age, and marital status) also affect individual’s job burnout [5, 14, 47]. Therefore, these variables were also measured and treated as control variables in this study. Socio-Demographic information consist of nine items which is gender, age, marital status, diploma level, social work age, yearly income, the nature of organization, number of employees and positional hierarchy.

### Statistical analysis

This study used SPSS 21.0 for Mac and Process for SPSS v2.13.2 for data analysis. All statistical tests were two sided (*α* = 0.05). Specially, descriptive statistics and one-way analysis of variance (ANOVA) were used to analyze HR managers’ job burnout status and differences in demographic variables, such as sex, age, marital status, educational background, and social work age. Correlation analysis was used to explore the relationship between listening competency, role stress, and job burnout of HR managers. Furthermore, regression analysis was conducted with job burnout as the dependent variable, and the predictive effects of listening competency and role stress on job burnout were tested so as to further explore the deep relationship between listening competency, role stress, and job burnout. Last but not the least, this study adopted the mediating effect analysis procedure proposed by Zhao et al. (2010) [48], referred to the Bootstrap method proposed by Preacher and Hayes (2004) [49], and used process plug-in provided by SPSS to test the mediating effect of role stress with a sample size of 5000 and a 95% confidence interval (Preacher et al., 2007) [50]. The significance of mediating effect was tested by confidence interval [51, 52].

## Results

Table 2 showed the result of descriptive statistical analysis. Overall, China’s HR managers have a low level of job burnout. Descriptive statistical results showed that 62.5% of HR managers scores were less than 50, and no obvious characteristic of job burnout existed. Moreover, 34.5% of HR managers showed mild burnout (scores between 50 and 75 points), and only 3.0% of HR managers showed serious job burnout (score greater than 75 points). Among the three dimensions of job burnout, emotional exhaustion had the highest score, which was widely considered the key component of burnout [53].

**Table 2:** Descriptive statistical analysis

	M ± SD	Less than 50	50 to 75	75 to 100	F
		62.5%	34.5%	3.0%	
Burnout	2.056 ± 1.013	1.422 ± .664	3.021 ± .324	4.000 ± .158	175.106***
EE	2.350 ± 1.360	1.730 ± 1.081	3.240 ± 1.045	4.900 ± .452	51.072***
DEP	1.996 ± 1.408	1.186 ± .878	3.203 ± .925	4.791 ± .941	113.935***
RPA	1.851 ± 1.215	1.322 ± 1.028	2.717 ± .989	2.722 ± .697	35.927***

*Note*: *** *P* < 0.001, **P < 0.01, *P < 0.05. EE represents emotional exhaustion, DEP represents depersonalization, RPA represents reduced personal accomplishment

Table 3 showed the result of One-way ANOVA. Specifically, women exhibited higher job burnout and lower personal achievement than men. From the distribution characteristics of age, HR managers aged between 21 and 30 years suffered from job burnout easily than those aged between 31 and 40 years. From the perspective of marital status, job burnout of unmarried HR managers was significantly higher than the burnout of those already married. As for educational background, HR managers with junior degree exhibited higher burnout and lower personal accomplishment. From the perspective of social work age, HR managers with longer social work ages were less prone to be burnout. Job burnout showed a significant downward trend with the increase in income level, positional hierarchy, and members of organization.

**Table 3:** The distribution characteristics of job burnout among HR managers on demographic variables

		JB (M ± SD)	EE (M ± SD)	DEP (M ± SD)	RPA (M ± SD)
Gender	Male	1.925 ± 1.049	–	–	1.571 ± 1.005
	Female	2.212 ± .951	–	–	2.184 ± 1.357
F		4.695*	–	–	15.540***
Age	21 to 30	2.297 ± .948	2.620 ± 1.352	2.250 ± 1.401	2.060 ± 1.210
	31 to 40	1.685 ± 1.018	1.910 ± 1.271	1.640 ± 1.341	1.52 ± 1.164
F		8.059***	5.819**	5.265**	4.224**
Marriage	Yes	1.667 ± 1.026	1.920 ± 1.326	1.590 ± 1.138	1.505 ± 1.184
	No	2.359 ± .900	2.680 ± 1.304	2.317 ± 1.350	2.122 ± 1.177
F		15.222***	9.400***	8.665***	8.015***
Education	Specialized	2.737 ± .630	–	–	3.075 ± 1.175
	Bachelor	2.053 ± .996	–	–	1.792 ± 1.155
	Master	1.737 ± 1.089	–	–	1.500 ± 1.146
F		6.884**	–	–	13.335***
Social work age (Year)	Less than 1	2.535 ± .898	2.760 ± 1.223	2.465 ± 1.481	2.398 ± 1.342
	1 to 3	2.400 ± .934	2.690 ± 1.445	2.340 ± 1.429	2.198 ± 1.258
	3 to 5	2.087 ± .782	2.410 ± 1.107	2.172 ± .933	1.759 ± .875
	5 to 10	1.784 ± .999	2.030 ± 1.307	1.711 ± 1.335	1.629 ± 1.015
	10 to 15	1.539 ± .992	1.950 ± 1.338	1.439 ± 1.456	1.268 ± 1.152
	More than 15	1.461 ± 1.224	1.690 ± 1.540	1.341 ± 1.357	1.348 ± 1.342
F		6.166***	2.910**	3.594**	4.683***
Yearly income (Thousand)	Less than 30	2.632 ± .879	2.720 ± 1.008	2.546 ± 1.294	2.617 ± 1.158
	30 to 80	2.333 ± 1.010	2.530 ± 1.400	2.414 ± 1.420	2.112 ± 1.366
	80 to 150	1.974 ± 1.001	2.290 ± 1.370	1.904 ± 1.417	1.762 ± 1.198
	150 to 300	1.908 ± .908	2.490 ± 1.414	1.750 ± 1.391	1.528 ± .853
	More than 300	1.240 ± .835	1.370 ± 1.130	1.100 ± .883	1.225 ± 1.026
F		7.537***	3.654**	4.933**	5.942***
Number of Employees	Less than 20	2.718 ± 1.032	–	–	3.022 ± 1.495
	20 to 100	2.346 ± .985	–	–	1.968 ± 1.177
	100 to 300	2.082 ± 1.066	–	–	1.809 ± 1.328
	300 to 1000	1.923 ± 1.068	–	–	1.883 ± 1.198
	1000 to 2000	1.760 ± .810	–	–	1.640 ± 1.049
	More than 2000	1.870 ± .905	–	–	1.473 ± .819
F		3.581**	–	–	6.124***
Rank	HR Specialist	2.338 ± .825	2.690 ± 1.256	2.398 ± 1.306	–
	HR Supervisor	1.935 ± .916	2.070 ± 1.171	1.776 ± 1.344	–
	HR Manager	1.772 ± 1.144	2.100 ± 1.488	1.712 ± 1.343	–
	HR Director	1.127 ± 1.022	1.160 ± 1.196	1.000 ± 1.486	–
	HRBP	1.690 ± .864	2.170 ± 1.573	1.429 ± 1.026	–
F		5.295***	4.070**	3.894**	–

*Note*: *** *P* < 0.001, **P < 0.01, *P < 0.05. JB represents job burnout, EE represents emotional exhaustion, DEP represents depersonalization, RPA represents reduced personal accomplishment

The mean value, standard deviation, and correlation coefficient of each variable are shown in Table 4. The results showed that listening attitude negatively correlated with job burnout and its three dimensions (*r* = − 0.341, *P* < 0.01; *r* = − 0.215, *P* < 0.01; *r* = − 0.333, *P* < 0.01; *r* = − 0.254, *P* < 0.01); listening skills negatively correlated with job burnout and its three dimensions (*r* = − 0.433, *P* < 0.01; *r* = − 0.333, *P* < 0.01; *r* = − 0.376, *P* < 0.01; *r* = − 0.303, *P* < 0.01); listening efficiency negatively correlated with job burnout and its three dimensions (*r* = − 0.299, *P* < 0.01; *r* = − 0.234, *P* < 0.01; *r* = − 0.273, *P* < 0.05; *r* = − 0.194, *P* < 0.01); and empathy negatively correlated with job burnout and its three dimensions (*r* = − 0.430, *P* < 0.01; *r* = − 0.304, *P* < 0.01; *r* = − 0.370, *P* < 0.01; *r* = − 0.327, *P* < 0.01). Listening attitude negatively correlated with role ambiguity (*r* = − 0.492, *P* < 0.01), but positively with role conflict (*r* = 0.139, *P* < 0.05). Listening skills negatively correlated with role ambiguity and role quality overload (*r* = − 0.542, *P* < 0.01; *r* = − 0.160, *P* < 0.05), and positively with role conflict and role load (*r* = 0.155, *P* < 0.05; *r* = 0.164, *P* < 0.05). Listening efficiency negatively correlated with role ambiguity and role quality overload (*r* = − 0.546, *P* < 0.01; *r* = − 0.148, *P* < 0.05). Empathy negatively correlated with role ambiguity and role quality overload (*r* = − 0.506, *P* < 0.01; *r* = − 0.197, *P* < 0.05), but positively with role conflict and role quantity overload (*r* = 0.185, *P* < 0.01; *r* = 0.159, *P* < 0.05). Role stress positively correlated with emotional exhaustion and depersonalization (*r* = 0.550, *P* < 0.01; *r* = 0.449, *P* < 0.01).

**Table 4:** Mean, standard deviation and correlation coefficient of each variable

Variable	Mean	S.D.	1	2	3	4	5	6	7	8	9	10	11	12	13
LA	5.384	.883	1.00												
LS	5.524	.877	.809**	1.00											
LE	5.102	1.002	.685**	.723**	1.00										
EM	5.742	.839	.746**	.813**	.636**	1.00									
RS	3.764	.697	−.282**	−.312**	−.397**	−.301**	1.00								
RC	5.168	1.270	.139*	.155*	−.067	.185**	.367**	1.00							
RA	2.639	1.047	−.492**	−.542**	−.546**	−.506**	.695**	−.403	1.00						
RQL_1_	3.771	1.222	−.120	−.160*	−.148*	−.197*	.728*	.159*	.233**	1.00					
RQL^2^	5.069	1.097	.114	.164*	.053	.159*	.486**	.239**	−.015	.262**	1.00				
JB	2.056	1.013	−.341**	−.433**	−.299**	−.430**	.472**	−.044	.448**	.464**	−.011	1.00			
EE	2.350	1.360	−.215**	−.333**	−.234**	−.304**	.550**	−.027	.402**	.506**	.248**	.774**	1.00		
DE	1.996	1.408	−.333**	−.376**	−.273*	−.370**	.449**	.032	.382**	.427**	.038	.845**	.637**	1.00	
RPA	1.851	1.216	−.254**	−.303**	−.194**	−.327**	.125	−.091	.264**	.165*	−.283**	.710**	.188**	.395**	1.00

*Note*: *** *P* < 0.001, **P < 0.01, *P < 0.05. LA represents listening attitude, LS represents listening skill, LE represents listening efficiency, EM represents empathy, RS represents role stress, RC represents role conflict, RA represents role ambiguity, RQL_1_ represents role quality overload, RQL_2_ represents role quantity overload, JB represents job burnout, EE represents emotional exhaustion, DEP represents depersonalization, RPA represents reduced personal accomplishment

The results of regression analysis are shown in Table 5. After controlling for demographic variables (such as gender and age), the results of regression analysis showed that listening attitude had a significant negative influence on emotional exhaustion, depersonalization, and reduced personal accomplishment (*β* = − 0.201, *P* < 0.001; *β* = − 314, *P* < 0.001; *β* = − 0.210, *P* < 0.001); listening skills had a significant negative effect on emotional exhaustion, depersonalization, and reduced personal accomplishment (*β* = − 0.294, *P* < 0.001; *β* = − 0.326, *P* < 0.001; *β* = − 0.223, *P* < 0.001); listening efficiency had a significant negative influence on emotional exhaustion, depersonalization, and reduced personal accomplishment (*β* = − 0.212, *P* < 0.001; *β* = − 0.234, *P* < 0.001; *β* = − 0.127, *P* < 0.05); and empathy had a significant negative influence on emotional exhaustion, depersonalization, and reduced personal accomplishment (*β* = − 0.267, *P* < 0.001; *β* = − 0.315, *P* < 0.001; *β* = − 0.238, *P* < 0.001). Role stress had a significant positive effect on emotional exhaustion and depersonalization (*β* = 0.510, *P* < 0.001; *β* = 0.395, *P* < 0.001); role conflict had no significant effect on job burnout; role ambiguity had a significant positive effect on emotional exhaustion, depersonalization, and reduced personal accomplishment (*β* = 0.364, *P* < 0.001; *β* = 0.332, *P* < 0.001; *β* = 0.201, *P* < 0.01); role quality overload had a significant positive effect on emotional exhaustion and depersonalization (*β* = 0.464, *P* < 0.001; *β* = 0.353, *P* < 0.001); role quantity overload had a significant positive effect on emotional exhaustion (*β* = 0.286, *P* < 0.001) and a significant negative effect on reduced personal accomplishment (*β* = − 0.200, *P* < 0.01). Therefore, both Hypothesis 1 and Hypothesis 2 were verified.

**Table 5:** The regression results of listening competency, role stress and job burnout

Variable	Burnout		EE		DEP		RPA	
	β	∆R^2^	β	∆R^2^	β	∆R^2^	β	∆R^2^
RS	.402***	.150***	.510***	.242***	.395***	.145***	.058	.003
RC	−.088	.007	−.070	.005	−.009	.000	−.112	.012
RA	.383***	.139***	.364***	.126***	.332***	.105***	.201**	.039**
RQL_1_	.364***	.121***	.464***	.196***	.353***	.113***	.054	.003
RQL_2_	.004	.068	.286***	.076***	.099	.009	−.200**	.037**
LA	−.037***	.091***	−.201**	.039**	−.314***	.095***	−.210***	.042***
LS	−.359***	.121***	−.294***	.081***	−.326***	.099***	−.223***	.047***
LE	−.242***	.057***	−.212***	.044**	−.234***	.053***	−.127*	.016*
EM	−.350***	.114***	−.267***	.066***	−.315***	.092***	−.238***	.052***

*Note*: *** *P* < 0.001, **P < 0.01, *P < 0.05. EE represents emotional exhaustion, DEP represents depersonalization, RPA represents reduced personal accomplishment, RS represents role stress, RC represents role conflict, RA represents role ambiguity, RQL_1_ represents role quality overload, RQL_2_ represents role quantity overload, LA represents listening attitude, LS represents listening skill, LE represents listening efficiency, EM represents empathy

Table 6 showed the result of mediating effect analysis. The results showed that 0 was not included in the test results of LLCI and ULCI, indicating that the mediating effect of role stress was significant (listening attitude: *b = − 0.1324, CI = [− 0.2069, − 0.0680*]; listening skills: *b = − 0.1344, CI = [− 0.1999, − 0.0788]*; listening efficiency: *b = − 0.1684, CI = [− 0.2431, − 0.1052]*; empathy: *b = − 0.1369, CI = [− 0.2018, − 0.0806]*). Therefore, role stress played a significant mediating role in the correlation between listening competency and job burnout, and it was partially mediating. Therefore, Hypothesis 3 was verified.

**Table 6:** The mediating effect of role stress on listening competency and job burnout

Model	Direct effect		Indirect effect	
	b	CI	b	CI
L	−.3690	[−.5158, −.2222]	−.1622	[−.2630, −.0959]
LA	−.2597	[−.3928, −.1266]	−.1324	[−.2069, −.0680]
LS	−.3659	[−.4970, −.2347]	−.1344	[−.1999, −.0788]
LE	−.1338	[−.2590, −.0086]	−.1684	[−.2431, −.1052]
EM	−.3824	[−.5188, −.2459]	−.1369	[−.2018, −.0806]

*Note*: b represents effect, CI represents Confidence Interval, L represents Listening Competency, LA represents Listening Attitude, LS represents Listening Skill, LE represents Listening Efficiency, EM Empathy

## Discussion

The results showed that 37.5% of HR managers experienced different levels of job burnout, and 3.0% had severe job burnout. China has undergone a rapid economic growth and radical social change over the past decades, which bring with them great psychological pressures that may translate into burnout [54]. In addition, the interview results showed that some organizations combine personnel and administration into one department in China, which results in a large work overload. HR managers are mostly engaged in transactional work; their work responsibilities are vague, and role stress becomes a common phenomenon. Existing studies have shown that role stress has a significant effect on job burnout [6, 55]. Fundamentally, HR managers themselves are also employees, so they can better understand the needs and difficulties of employees in work. Therefore, HR managers are easier to produce empathy fatigue, which can lead to job burnout [56]. As a result, HR managers are facing a severe crisis of job burnout. Since the beginning of the twenty-first century, China’s social and economic development level has increased rapidly. However, the basic situation that China is still in the primary stage of Socialism has not changed. Therefore, the job burnout of HR managers in China is still at a low level.

Similar to previous studies [32, 57], we found that demographic variables are related to job burnout. The one-way ANOVA results showed that female HR managers have a higher level of job burnout and a lower sense of personal accomplishment than male HR managers, which was consistent with the results of Xie et al. (2020) [58]. And according to the Chinese tradition, women need to take more family responsibilities than their husbands. Bu & Mckeen (2000) [59] have found that Chinese women spend an average of 3.7 h on housework every day, while men only spend 2 h. In addition, Pinto et al. (2014) [60] found that women suffer a higher risk of burnout due to work-life imbalance, occupational discrimination and multiple social roles. Therefore, under the dual pressure and conflict between work and family, female HR managers are more prone to suffer job burnout. From the perspective of age, HR managers aged between 21 and 30 years have a significantly higher level of job burnout than those aged between 31 and 40 years, which was consistent with the results of (Chambers, 2016; Lu et al., 2020) [61, 62]. And was not consistent with the results of Karatepe and Uludag (2008) [63], which indicated that age and education have positive relationships with job burnout. First, this can be explained by the classical JD-R model, most HR managers aged between 21 and 30 years are university graduates, they work in the society for a short time and lack of experience and resources to cope with demanding situations at work, which was associated with depersonalization and reduced personal accomplishment. Second, traditional Chinese parents generally believed that “A man should get married on coming of age, and so should a girl”. University graduates face their parents’ forced marriage immediately after entering the workplace. Also, HR managers aged 31–40 years have longer working time and a stable family, as they are in the rising stage of their career. Therefore, HR managers aged 21–30 years have a higher level of job burnout than those aged 31–40 years. The same reason can also explain the higher levels of job burnout among unmarried HR managers and those with shorter working hours. From the perspective of educational background, when the education level increases, the job burnout level of HR managers tends to decrease. The highly educated HR managers have higher professional skills, can better deal with various job problems, and have higher work efficiency. This was consistent with the results of Rashkovits and Livne (2013) [64], they indicated that highly educated employees may be more rational and calmer, which may help them manage burnout. Second, education level often has a positive correlation with their salary in China. According to the Effort–Reward model [55, 65], HR managers with a lower income level are more likely to feel that they are not fairly paid for their efforts, and thus more likely to get a job burnout. The interview results showed that small organizations always lacked professional HR management teams, resulting in heavy work overload of HR managers and excessive consumption of personal resources. According to the JD-R model, when HR managers fail to meet excessive work requirements, job burnout occurs. Finally, a lower-level position means more work tasks and lower salary in China. Meanwhile, most HR managers with a lower position are college students who have just graduated from university and have fewer work resources, so they are more likely to suffer a job burnout.

Correlation analysis and regression analysis confirmed the Hypothesis 1 that HR managers who are good at listening were more likely to avoid job burnout. Listening competency is a special skill [66]. Ames et al. (2012) [18] found that HR managers who are good at listening were more influential, and listening skills were also an important factor that motivated people to achieve the desired behavioral results [67]. Therefore, HR managers who are good at listening not only gain advantages in developing relationship but also have increased self-confidence and respect [68]. Moreover, effective listening is a prerequisite for good service and interaction. It is also conducive to creating a communication atmosphere of mutual understanding and support between HR managers and their leaders or employees to improve work performance. Studies have shown that individual-oriented interventions are more effective than organization-oriented interventions, and that interventions tailored to employees’ preferences and abilities may help prevent job burnout. Through job shaping, employees optimize their work needs and resources, and increase their personal resources [69, 70]. According to the JD-R model [39], HR managers who are good at listening can easily establish a good interpersonal relationship with employees, accumulate rich network interpersonal resources, improve work efficiency, and optimize the quality of HR management, thereby reducing their level of job burnout [5, 6, 71].

The results also confirmed that role stress and job burnout are significantly and positively related. Thus, the higher the degree of role stress, the higher the incidence of burnout, which was also observed in previous studies [6, 25, 32, 72]. However, not every dimensions of role stress have a significant correlation with job burnout of HR managers. Among which, role conflict had no significant effect on job burnout, as well as role ambiguity had a significant positive effect on job burnout, and this was not consistent with the previous studies [32, 72]. On the one hand, as HR managers said in the interview, role conflict is one of the unavoidable aspects of their work. Under this circumstance, HR managers might not have had negative self-evaluations when they had received incompatible demands form multiple sources such as leaders and employees. On the other hand, when HR managers are ambiguous about his or her various roles–whether due to their own or external factors – they lack a clear understanding of their roles, work content and purpose. Therefore, the ambiguity of their roles will lead to the uncertainty of their own work. This uncertainty makes HR managers constantly spend their own resources on seeking and obtaining related information. When HR managers do not receive additional resources, there will be an imbalance between resources and needs, which in turn can easily lead to job burnout [73]. These findings indicate that leaders should attempt to reduce role stress in HR managers and prevent job burnout, such as reducing HR managers’ work overload and improving their working conditions.

In addition, the results of mediating effect analysis showed that role stress played a significant mediating role in the correlation between listening competency and job burnout, and it was partially mediating. This can be explained by two mechanisms. On the one hand, as HR managers said in the interview, their role stress mainly comes from their contradictory work expectations and excessive work responsibilities. Kazu and Demiralp (2015) [36] suggested that people acquire most their knowledge by listening, that is to say, HR managers can acquire more knowledge and skills of HR management through listening, thus improving work efficiency, reducing role stress and ultimately reducing the risk of job burnout. In addition, listening was also a method to solve psychological distress. Active listening was also regarded as an important factor to reduce anxiety and pain. Effective listening can reduce HR managers’ psychological conflicts caused by role stress, and ultimately reduce role stress and job burnout. On the other hand, good listening competency can help HR managers grasp the real needs and expectations of leaders and employees, so as to better balance the interests between enterprises and employees, management and service, and institution and human relationship. Good listening competency also helps HR managers to clarify their responsibilities and decrease unnecessary resource consumption, which also can reduce their role stress and job burnout. In other words, HR managers with good listening competency can reduce their job burnout by reducing their role stress. Hence, Hypothesis 3 was verified.

A strong point of this study is that Chinese HR managers’ job burnout has been surveyed, which has extended the knowledge in burnout research. Moreover, to our knowledge, this was the first study to introduce listening competency into the field of job burnout research. With the correlation analysis, regression analysis and mediating effect analysis, the relationships among listening competency, role stress and job burnout were explored. Furthermore, this study also has several limitations. First, convenience sampling, use of Internet for survey distribution, and the number of survey items may have impacted response rates. And data were collected by self-report questionnaire, the self-report nature of the measures leaves open the possibility that reported information may not accurately reflect the underlying values of each variable, which would have biased the data. Second, this study was a cross-sectional design, however, job burnout changes over one’s employment period [74]. Thus, a longitudinal study is necessary for future research. Third, although a representative sample was selected, sample weight calculation was not considered in this study and therefore the extrapolation of conclusions would be limited to some extent. Finally, there may be numerous important aspects related to burnout that were not measured in this study, such as geographic and industry factors, there is a need for ongoing studies to explore these factors in practice. Despite these limitations, this study provides a glimpse into the listening competency and job burnout of HR managers in China for future research.

## Conclusion

More than one third of the HR managers reported mild burnout or serious burnout, which indicated that the job burnout among HR managers in China organizations cannot be ignored. And among them, emotional exhaustion was the most serious, followed by the depersonalization, and the lowest was reduced personal accomplishment. In addition, listening competency had a significantly negative impact on role stress and job burnout, role stress had a significant positive impact on HR managers’ burnout, and there may be partial mediation effects of role stress within the impact of listening competency on job burnout among HR managers. In other words, listening competency can reduce job burnout by reducing the role stress of HR managers. These findings signify that leaders should pay more attention to HR managers’ job burnout and the risks resulting from it. Efforts should be made to develop strategies to reduce role stress and to strengthen listening competency, thereby mitigating the risk of job burnout.

## Data Availability

The datasets used and/or analysed during the current study are available from the corresponding author on reasonable request.

## Abbreviations

ANOVA: Analysis of Variance
HR: Human Resource
JD-R: Job Demand–Resource model
MBI-GS: Maslach Burnout Inventory-General Survey
